# An Example of Family Tabes Dorsalis

**Published:** 1905-09-16

**Authors:** 


### AN EXAMPLE OF FAMILY TABES DORSALIS.

Dr. E. F. Trevelyan[1] relates the following family record:—A man who married when aged 22 years showed early symptoms of tabes eleven years later, and subsequently became markedly ataxic; he died aged 58. His wife, who was 19 when she married, developed tabetic symptoms at 29 years and, later, suffered from attacks of vomiting and bladder symptoms with arthropathy of each knee-joint; at the present date the disease appears to be stationary. The eldest daughter, now aged 43 years, complained of pains and occasional double vision when 36, and since has manifested such characteristic symptoms as loss of the knee- and heel-jerks, Argyll-Robertson pupils, girdle sensation, and zone-anæsthesia of the trunk. There was no satisfactory evidence of past syphilis in the parents, but such a conclusion is indicated both by the occurrence of tabes and by the family history in the second generation. The daughter married and had five pregnancies; there was no reason to believe that either she or her husband suffered from acquired syphilis.

Conjugal tabes has been recorded on many occasions. Usually the disease begins in the husband and the wife develops symptoms some years later. Tabes in husband, wife, and offspring is extremely exceptional. Cases of tabes in one parent and in the offspring are comparatively numerous. Dr. Trevelyan gives a very full bibliography bearing on all these questions, and many of the records quoted are of great value. The article should be consulted by all who are interested in the subject.

[1] Lancet, Sept. 9, 1905.

